# Human Bone Marrow Contains Mesenchymal Stromal Stem Cells That Differentiate In Vitro into Contractile Myofibroblasts Controlling T Lymphocyte Proliferation

**DOI:** 10.1155/2018/6134787

**Published:** 2018-04-29

**Authors:** Yves Lecarpentier, Olivier Schussler, Antonija Sakic, José Maria Rincon-Garriz, Priscilla Soulie, Marie-Luce Bochaton-Piallat, Vincent Kindler

**Affiliations:** ^1^Centre de Recherche Clinique, Grand Hôpital de l'Est Francillien, 77104 Meaux, France; ^2^Department of Cardiovascular Surgery, Research Laboratory, University Hospitals, Faculty of Medicine, Geneva, Switzerland; ^3^Department of Pathology and Immunology, Centre Médical Universitaire Geneva Faculty of Medicine, Geneva, Switzerland; ^4^Department of Specialties in Medicine, Hematology Service, Geneva University Hospitals, Faculty of Medicine, Geneva, Switzerland; ^5^Department of Histology, Centre Médical Universitaire Geneva Faculty of Medicine, Geneva, Switzerland

## Abstract

Mesenchymal stromal stem cells (MSC) that reside in the bone marrow (BM) can be amplified in vitro. In 2-dimension (D) cultures, MSC exhibit a morphology similar to fibroblasts, are able to inhibit T lymphocyte and natural killer cell proliferation, and can be differentiated into adipocytes, chondrocytes, or osteoblasts if exposed to specific media. Here we show that medullar MSC cultured in 2D formed an adherent stroma of cells expressing well-organized microfilaments containing *α*-smooth muscle actin and nonmuscle myosin heavy chain IIA. MSC could be grown in 3D in collagen membranes generating a structure which, upon exposition to 50 mM KCl or to an alternating electric current, developed a contractile strength that averaged 34 and 45 *μ*N/mm^2^, respectively. Such mechanical tension was similar in intensity and in duration to that of human placenta and was annihilated by isosorbide dinitrate or 2,3-butanedione monoxime. Membranes devoid of MSC did not exhibit a significant contractility. Moreover, MSC nested in collagen membranes were able to control T lymphocyte proliferation, and differentiated into adipocytes, chondrocytes, or osteoblasts. Our observations show that BM-derived MSC cultured in collagen membranes spontaneously differentiate into contractile myofibroblasts exhibiting unexpected properties in terms of cell differentiation potential and of immunomodulatory function.

## 1. Introduction

Mesenchymal stromal stem cells (MSC) can be derived from almost any vascularized tissue of the organism after in vitro amplification with media supplemented with foetal calf serum, platelet-derived growth factor (PDGF), or human platelet lysate (HPL) [1–4]. The identification of the in vivo counterpart of MSC has been rather difficult to achieve because MSC precursors are scarce in vivo [5, 6]. For this reason in vitro amplification of MSC is generally required prior to any investigation. Since the mean doubling time of MSC derived from BM cultured with PDGF is 4 days [6], cells that are investigated at passages 2-3 (i.e., 20 days after seeding) have already achieved a significant number of mitosis in vitro and maybe biased compared to their in vivo counterpart. Nevertheless, there is now a growing consensus based on similarities in cell surface marker expression and in biological functions that MSC are derived from pericytes and/or adventitial cells that encircle the microvasculature of vascularized organs and tissues [7–9]. The media used to amplify MSC in vitro contain various biologically active factors including transforming growth factor-*β* (TGF-*β*), PDGF, basal fibroblast growth factor, and insulin-like growth factor-1 which display proinflammatory features in specific situations. In particular TGF-*β* and PDGF that are released upon platelet activation participate to the migration of quiescent pericytes to inflammatory sites where they differentiate into myofibroblasts and provide a healing activity (for a review, see [10–12]). Although the environment required to achieve MSC amplification in vitro holds similarities with that observed in vivo when pericytes are activated after tissue injury, the hyperoxia produced by atmospheric oxygen, the artificial plastic surface lacking key adhesion molecules, and the repetitive thermic shocks and trypsinations may bias cell differentiation compared with the in vivo situation. The cells obtained after in vitro amplification are plastic adherent, prolong quiescent B and T lymphocyte survival, and differentiate into various mesenchymal lineages if exposed to the appropriate stimulus. Moreover, when exposed to an additional inflammatory stimulus such as *γ*-interferon (*γ*-IFN), MSC prevent T lymphocyte, B lymphocyte, or natural killer (NK) cell proliferation [13–15], thus demonstrating anti-inflammatory properties. Concerning human MSC, one of the major factors mediating T cell control is the enzyme indoleamine 2,3-dioxygenase (IDO), which degrades the essential amino acid L-tryptophan (tryp) in L-kynurenine (kyn) [16–19]. IDO is a holoenzyme containing a heme moiety that is functional only when the heme is reduced in the ferrous form by yet unidentified cofactors [20]. Consequently, the detection of either IDO mRNA or protein does not prove that the enzyme is functional; rather, the detection of the product metabolized by the enzyme (i.e., kyn) is required for such a claim. IDO is activated in MSC by various proinflammatory factors including *γ*-IFN, which is provided upon the course of an inflammatory process by the early activation of T lymphocytes and NK cells (see [18]). lDO can degrade tryp to such an extent that T lymphocyte, B lymphocyte, and NK cell proliferation, whose initiation and sustenance rely upon tryp concentration, is either prevented or arrested [16–19].

Myofibroblasts are contractile cells initially identified in the skin [21]. They express *α*-smooth muscle actin (*α*SMA) and the nonmuscle myosin heavy chain IIA (NMMHCIIA) that are both required to achieve cell contraction. Upon injury occurrence, myofibroblasts synthesize extracellular matrix proteins and retract, which favors healing and injury resolution [22]. Moreover, some myofibroblasts exhibit anti-inflammatory properties and may also favor injury resolution by shutting down inappropriate delayed immune response [23]. The ancestor of the myofibroblast is not fully identified yet. Fibrocytes, pericytes, and tissue resident fibroblasts have all been pointed out as possible progenitors of these cells [24, 25], and recently MSC derived from neonatal lungs have been shown to express a gene profile consistent with a myofibroblast progenitor phenotype [26], further emphasizing the similarities between MSC and myofibroblasts. It was therefore of interest to investigate whether myofibroblasts could be generated from a tissue such as bone marrow that is commonly used to generate MSC.

In order to do so, we designed a 3D culture system that allowed to maintain cells in a biocompatible scaffold for an extended period of time, and we developed specific devices to measure the mechanical strength generated by the cells immobilized in the scaffold. MSC were derived from BM, HPL was used as growth factor, and the scaffold consisted in 6 mm diameter patches of commercially available bovine collagen membranes.

We show here that under these culture conditions MSC express markers that are identical to that of 2D cultures including *α*SMA and NMMHCIIA, and that the cells contract and develop a sizable mechanical tension when exposed to relevant stimuli. Moreover, once resident in the membranes, MSC maintain their ability to differentiate into various mesenchymatous lineages and exert a strong control upon T cell proliferation. Thus MSC amplified in vitro exhibit features of both MSC and myofibroblasts, which allows wondering whether these cells are close parents.

## 2. Materials and Methods

### 2.1. Reactants

#### 2.1.1. Cytokines and Chemicals

1,4-Dithio-DL-threitol (DTT), 3-isobutyl-1-methylxanthine (IBMX), indomethacin (Indo), pyruvic acid, L-ascorbic acid 2-phosphate, dexamethasone, insulin, chondroitinase-ABC sodium *β*-glycerophosphate (*β*GP), and ascorbic acid (AsA) were from Sigma-Aldrich Chemie GmbH, Schnelldorf, Germany. L-Ascorbic acid-2-phosphate (AsAP) was purchased from Wako Pure Chemical Industries, Ltd. (Osaka, Japan), 1,25(OH)_2_ vitamin D3 (vitaminD3) from BIOMOL (Plymouth Meeting, MA), and TRI Reagent from Molecular Research Center (Cincinnati, OH). ITS+ insulin, transferrin, selenious acid, bovine serum albumin, and linoleic acid premix (ITS) were from BD Biosciences, San Diego, CA. TGF-*β* was from R&D Systems. Hank's balanced salt solution (HBSS), Roswell Park Memorial Institute (RPMI) 1640 medium, Iscove's modified Dulbecco's medium (IMDM), and penicillin-streptomycin trypsin-EDTA solution were from Gibco BRL, Paisley, UK. 5(and 6)-carboxy fluorescein diacetate, succinimyl ester (CFSE) was from Molecular Probes Europe BV, Leiden, Netherlands. Collagenase type II and 4′,6-diamidino-2-phenylindole dihydrochloride (DAPI) were from Sigma-Aldrich. 3,3′-diamino-benzidine chromophore was from DakoCytomation, Glostrup, Denmark. Streptavidin-peroxydase kit (Vectastain, ABC kit, BA2000) was from Vector Laboratories, Burlingame, CA, and 3,3′-diaminobenzidine tetrahydrochloride dihydrate (DAB) from Fluka Chemie.

#### 2.1.2. Antibodies

*(1) FITC Labeled.* Anti-CD54 (clone 15.2 mouse (m) IgG1) and anti-CD31 (clone 158-2B3 mIgG1) were from Ancell Corp., Bayport, MN, anti-CD45 (clone J33 mIgG1) was from Immunotech, Marseille, France, anti-CD90 (clone 5E10 mIgG1) was from BD Biosciences, San Diego, and anti-HLA-ABC (clone G46-2.6 mIgG1) was from BD Biosciences.

*(2) RPE Labeled.* Anti-CD3-RPE (clone UCHT-1, mIgG1) was from Ancell Corp., anti-CD34 (clone 8G12 mIgG1) was from BD Biosciences, anti-CD105 (clone SN6 mIgG1) was from AbD Serotec, Oxford, UK, anti-CD146 (clone P1H12 mIgG1) was from BioLegend, San Diego, CA, and anti-HLA-DR (clone 581 mIgG1) was from BD Biosciences.

*(3) APC Labeled.* Anti-CD44 (clone IM7 mIgG2b) was from eBioscience, anti-CD56 (clone AF12-7H3 mIgG1) was from Myltenyi Biotec, and anti-CD140b (clone 18A2 mIgG1) was from BioLegend.

*(4) Unlabeled.* Anti-*α*SMA (clone 1A4 mIgG1) was from [27].

*(5) Monoclonal Isotype Controls*. FITC, PE and biotin-labeled mIgG1, and biotin-labeled mIgG2b were from Dako, biotin-labeled mIgG2b was from Ancell, and unlabeled mIgG1 (clone MOPC-21) was from BioLegend.

*(6) Polyclonal Antibodies.* Anti-SMMHCs types 1 and 2 rabbit polyclonal IgG (BT-562) was from Biomedical Technologies Inc., Stoughton, MA, and anti-nonmuscle myosin heavy chain IIA (NMMHCIIA) rabbit polyclonal IgG (MYH9) was from Sigma-Aldrich.

*(7) Secondary Antibodies.* FITC-conjugated goat anti-mIgG2a and rhodamine-conjugated goat anti-rabbit IgG used as secondary antibodies were from Molecular Probes.

### 2.2. MSC Purification, Amplification, and In Vitro Differentiation

#### 2.2.1. Purification

Cells were prepared and amplified in 2D cultures as previously reported [6]. Femoral heads were collected during hip replacement surgical interventions, according to the guidelines of the local ethics committee and after the patient's informed consent. Briefly, the fatty pulp of the femoral heads was released by scraping the bone with a spoon-shaped curette and deposited in a 50 ml Falcon tube containing Hank's balanced salt solution. The bone debris were allowed to settle for few minutes, and the cell suspension was transferred in a fresh tube. The sedimentation procedure was repeated once. Then a sample was collected, red cells were lysed with a hypotonic NH_4_Cl solution, and nucleated cells were counted with a Neubauer hemocytometer. One million of live (trypan blue excluding) cells were distributed in 100 mm diameter Petri dishes containing 10 ml of RPMI medium complemented with 5% HPL and heparin. This led to final concentrations of 2.5–6 ng/ml of TGF-*β*, 0.5–1.5 ng/ml of PDGF, 4.5–9.5 pg/ml of basic fibroblast growth factor (*β*FGF, also known as FGF-2), and 5.3–6.5 ng/ml of insulin growth factor like (IGF) [3, 28]. After 48 hours, nonadherent cells were washed out and cultures were fed with the initial medium until subconfluency. Cells were then trypsinated (passage 1) and seeded at 10^5^/100 mm diameter Petri dish (corresponding to 1.3 × 10^3^ cells/cm^2^) and grown until at half confluence (4 × 10^3^/cm^2^). Cultures up to passage 4 were used for the experiments.

#### 2.2.2. MSC Seeding in Collagen Membranes

Small cylinders of Avitene™ Ultrafoam™ collagen hemostat (Bard Limited, Crawley, UK, ref. 1050050, 20–200 *μ*m pore diameter, bovine dermal collagen of type I) were obtained by punching the commercially available membrane humidified with PBS with a sterile dermatologic biopsy punch of 6 mm diameter (Integra Miltex, Fisher Scientific). Each individual specimen, referred thereafter as a “membrane”, was soaked for at least 24 hours in RPMI 5% HPL containing heparin. Membranes were then laid on a sterile gauze, and 10^5^ MSC in 10 *μ*l were delicately deposited on their upper surface. Cells were allowed to penetrate the patch for 1 hour at 37°C. The membranes were transferred in wells of a 24-well culture plate, containing 500 *μ*l of medium. Medium was exchanged twice a week.

*(1) Membrane Surface Measurement.* Wet membranes loaded or not with MSC were laden on and photographed over a sterile petri dish whose basement was gridded with 4 mm^2^ squares. Using the square grid, maximum and minimum diameters of each membrane were determined. These values were converted in mm, and one was multiplied by the other to obtain the approximate membrane surface.

*(2) MSC Release.* Membranes were removed from the culture vessel, transferred in Eppendorf conical tubes containing 1 ml of Hanks balanced salt solution (HBSS), and washed twice with HBSS. Digestion was undertaken in 1000 *μ*l of a solution of HBSS supplemented with 3 mM CaCl_2_ and 125 U/ml collagenase II, for 10 minutes at 37°C with strong vortexing at 5-minute intervals, or until full dissolution of the membrane. The cell suspensions were then centrifuged for 10 seconds at full speed on a Hettich table centrifuge and pellets were resuspended in 100 *μ*l of RPMI 10% FCS prior to cell counting with the hemocytometer.

#### 2.2.3. MSC In Vitro Differentiation

*(1) Chondrogenic Differentiation.* Chondrogenic differentiation was induced as previously described [29]. Four days after seeding, membranes were transferred in serum-free DMEM with 1% ITS+, 1 mM pyruvic acid, 37.5 *μ*g/ml L-ascorbic acid-2 phosphate, 10^−7^ M dexamethasone, and 10 ng/ml TGF-*β*. Cells were cultured for 2 to 4 weeks, with medium changes every 3 days. Membranes were fixed in 4% paraformaldehyde, embedded in paraffin and cut into 5-micron thick sections. Sections were stained with Goldner's solution. Membranes from cultures in RPMI plus HPL were used as negative controls.

*(2) Adipogenic Differentiation*. Adipogenic differentiation was undertaken according to [5]. Briefly, 4 days after MSC seeding, membranes were incubated in the induction medium consisting of low glucose (1 g/l) DMEM, 10 *μ*g/ml insulin, 10^−6^ M dexamethasone, 5 × 10^−4^ M IBMX, and 10^−4^ M indomethacin. After 48 hours, this medium was washed out and replaced by the maintenance medium consisting of high glucose (4.5 g/l) DMEM, 10% horse serum (HS), and 10^−6^ M dexamethasone for 48 hours. This cycle was repeated 3 times. Membranes were then cultured in the maintenance medium until analysis. 2D cultures were established simultaneously as 3D cultures and submitted to the adipogenic differentiation procedure to determine the best time for adipocyte detection on the membranes. In general adipocytes were detectable in the 2D cultures 3 weeks after differentiation induction. Once the presence of adipocytes in the 2D cultures was confirmed, the corresponding membranes were then cryopreserved, sliced on a microtome, and stained with oil Red O.

*(3) Osteoblastic Differentiation.* Osteoblastic differentiation was achieved according to [30]. Four days after membrane seeding, medium was shifted to DMEM complemented with 10 × 10^−7^ M dexamethasone, 10^−2^ M*β*GP, and 5 × 10^−5^ M AsAP for 3 weeks. Membranes where then embedded in paraffin and cut into 5-micron thick sections prior to staining with Alizarin Red S.

### 2.3. Regulatory Activity of MSC upon Allogenic T Lymphocyte Proliferation

CD3+ T lymphocytes were obtained from peripheral blood of healthy donors after Ficoll-Paque gradient and negative selection (Dynal Biotech ASA, Oslo, Norway) and were labeled with 80 nM of CFSE [31] prior to coculture with allogenic MSC. MSC were seeded at 5 × 10^4^ cells in 24-well plates in 500 *μ*l of RPMI supplemented with 5% HPL and heparin 24–48 hours before the assays. Medium was replaced by fresh RPMI containing 10% FCS with 5 × 10^4^ allogenic T lymphocytes per well. Alternatively MSC-laden or empty membranes were transferred into wells containing fresh RPMI, FCS, and 5 × 10^4^ allogenic T lymphocytes. Cocultures were complemented with beads coated with anti-CD3 and anti-CD28 antibodies (a3-28) (Dynal Biotech) in a ratio of 0.5 beads per T lymphocyte seeded. Cell proliferation was assessed by flow cytometry after 5 days of coculture with MSC.

### 2.4. L-Tryptophan and L-Kynurenine Detection

Tryp and kyn concentrations were determined a described in [32, 33], respectively. Tryp was determined by fluorescence of the cleared culture supernatants after exposure to formaldehyde and FeCl_3_ on a Twinkle LB 970 plate fluorimeter (Berthold Technologies, Regendsorf, Switzerland) with *λ*_ex_ = 355 nm and *λ*_em_ = 460 nm. Kyn was determined by absorbance of the culture supernatant at 490 nm on a Vmax ELISA plate reader (Molecular Devices Corporation, Menlo Park, CA) after precipitation with trichloroacetic acid and incubation with Ehrlich's reagent.

### 2.5. XTT Assay

The XTT assay was performed according to the manufacturer's instructions. Membranes recovered from the various incubations at 4°C were placed in 24-well plates containing 200 *μ*l of fresh RPMI complemented with HPL and heparin. 100 *μ*l of XTT (2,3-bis-(2-methoxy-4-nitro-5-sulfophenyl)-2H-tetrazolium-5-carboxanilide) was added to each well containing a membrane. After a 2-hour incubation at 37°C, 200 *μ*l of supernatant was removed and the absorbance was measured at *λ* = 450 nm using an ELISA plate reader with a reference at *λ* = 650 nm. Membranes were then washed and photographed before resuming culture.

### 2.6. Immunofluorescence and Immunohistochemistry

#### 2.6.1. Immunofluorescence

MSC were fixed for 20 minutes in RPMI 1% paraformaldehyde (PFA), 2% HEPES, rinsed in PBS, and incubated for 5 minutes at −20°C in methanol prior to staining with anti-*α*SMA [34]. Alternatively cells were fixed in ethanol for 30 seconds and stained with anti-SMMHCs or anti-NMMHCIIA. Whole membranes were fixed for 45 minutes in PBS 1% PFA, rinsed in PBS, and further incubated for 15 minutes at −20°C in methanol prior to staining with anti-*α*SMA. In some instances slides were fixed in ethanol for 1 minute and stained with anti-SMMHCs or anti-NMMHCIIA. Slides were mounted in buffered polyvinyl alcohol.

#### 2.6.2. Immunohistochemistry

Membranes were fixed in 4% PFA, embedded in paraffin, and cut into 5-micron thick sections. Immunostaining for *α*SMA, SMMHCs, and NMMHCIIA were performed as described in [34, 35]. Sections were exposed to microwave radiation (750 W, 5 minutes) in citrate buffer (10 mM, pH 6.0) for *α*SMA and to a pressure cooker (3 minutes) in citrate buffer for SMMHCs and NMMHCIIA. Goat anti-mouse- or anti-rabbit-biotinylated IgGs were used as second antibodies. For visualization, the streptavidin-biotin peroxidase complex and 3,3′-diamino-benzidine chromophore was employed. Hemalun was used as counterstaining.

Double immunofluorescence staining of paraffin sections was also performed [34]. Slides were exposed to microwave radiation (250 W, 20 minutes) in Tris/EDTA (10 mM/1 mM, pH 9.1); specimens were then double stained with anti-*α*SMA and anti-SMMHCs or NMMHCIIA. FITC-conjugated goat anti-mIgG2a and rhodamine-conjugated goat anti-rabbit IgG were used as secondary antibodies. Nuclei were stained with DAPI. Slides were mounted in buffered polyvinyl alcohol. Images were recorded on an Axioskop 2 microscope (Carl Zeiss, Jena, Germany) equipped with a plan-Neofluar ×20/0.50 objective or an oil immersion plan-Neofluar ×40/1.4 objective and a high sensitivity, high-resolution digital color camera (Axiocam, Carl Zeiss). Whole membrane images were acquired using a confocal laser scan microscope (LSM800 Airyscan, Carl Zeiss) equipped with 2 lasers (excitation wavelengths at 488 and 561 nm) through an oil immersion Plan-Apochromat 63x/1.40 DIC f/ELYRA objective. Contrast and color adjustment of pictures, when required, were done using Adobe PhotoShop and applied across the integrality of the images. Similar levels of corrections were applied on the controls and the specimens labelled with the specific antibodies.

### 2.7. Electron Microscopy

MSC-seeded membranes were fixed with 1.5% glutaraldehyde in 0.1 M sodium cacodylate (Merck, Darmstadt, Germany) containing 1% sucrose for 3 hours. This was followed by fixation in 1% osmium tetroxide for 1 hour and subsequent dehydration and embedding in Epon. Semithin sections were stained with toluidine blue. Thin sections were treated with uranyl acetate and lead citrate and examined in an electron microscope (Philips CM10 TEM; Philips, Eindhoven, the Netherlands).

### 2.8. Determination of the Contractility of MSC Seeded in Collagen Membranes

Settings for the monitoring of the mechanical study were performed as previously described [36]. Briefly, empty or MSC-laden membranes obtained after 19 days of culture that were cooled to 4°C for 48 to 72 hours before processing in the experiments were used (such a delay was unavoidable due to the shipment from the laboratory where MSC were grown to the location were contractibility experiments were undertaken). Twenty-five membranes laden with MSC and 23 empty membranes were studied. The membrane mechanical parameters were controlled by the software running on the recording PC. The lever displacement that mirrored membrane contraction was measured by means of an optoelectronic transducer. A small diaphragm on the lever modulated the light of a light-emitting diode (LED) falling on a photodiode. The transducer was constructed from a suitable d'Arsonval panel meter whereby the pointer was replaced by a thin L-shaped stainless steel tube acting as the lever.

#### 2.8.1. Initial Settings

Under a binocular microscope, one end of the membrane was held by a small artery clip (S&T B-1 clamp) and fixed to a small linear micrometric translation stage. The opposite end of the membrane was then fixed to the tip of a homemade force-length transducer that was disposed in such a way that the membrane remained horizontal. Membranes were allowed to settle for 30 minutes at room temperature in a Krebs-Henseleit solution bubbled with 95% O_2_-5% CO_2_. The initial position for the displacement range was adjusted with a micrometer screw to a preload value of about 0.2 milliNewtons (mN). Basal resting tone (BT in mN/mm^2^), which was the tension where neither spontaneous shortening nor lengthening of the matrix occurred, and initial length (Li) were recorded.

#### 2.8.2. Membrane Passive Properties and Young's Modulus Computation

Membranes were then submitted to progressive elongation (in the absence of any other stimulus) to determine the Young modulus. The Young modulus (*E*) represents the slope of the stress/strain relationship. Stress (*σ*) is the force (*F*) per unit cross-sectional area (in m^2^) (in Pascal (Pa) or N/m^2^) imposed to the membrane and is expressed as

(1) $$\begin{array}{c} \sigma =\frac{F}{CSA}. \end{array}$$

Strain (*ε*) is the deformation of the membrane due to stress, that is, the elongation divided by the diameter of the membrane (*L*) at rest and is expressed as *ε* = dL/*L*, where dL is the elongation of the membrane. The Young modulus *E* is expressed as *E* = stress/strain (expressed in Pascal; Pa).

(2) $$\begin{array}{c} E=\frac{F/CSA}{dL/L}. \end{array}$$

Twenty-five membranes laden with MSC and 23 empty membranes were studied.

#### 2.8.3. Induction of Contraction

All membranes (laden with or devoid of MSC) were exposed either to alternating electric tetanus (train period: 5 seconds; train duration: 2 seconds; stimulus frequency: 100 Hz) or to 50 mM KCl, until reaching a plateau corresponding to the maximum amplitude of isotonic shortening (Δ*L*_max_). The membranes were then abruptly submitted to an isometric condition to measure the total isometric tension (TT). We measured the maximum amplitude of isotonic shortening (Δ*L*_max_) and the total isometric tension. At the end of the experiment, membrane weight and diameter were measured to calculate their effective cross-sectional area (CSA in mm^2^) using the ratio of the effective membrane weight (total weight/3) and the membrane diameter. The force observed (in mN) was normalized per CSA leading to tension (in mN/mm^2^). The active tension was calculated as the total tension minus BT. Shortening length was normalized by Li (% Li).

### 2.9. Statistics

Mann–Whitney nonparametric rank test was used when 2 sets of data were compared and the Gaussian distribution could not be assessed. A value of *p* < 0.05 was considered as significant. For multiple comparisons, ANOVA with the Bonferroni correction was used after confirmation of the Gaussian distribution of the data. Means ± SDs are shown if not stated differently.

## 3. Results

### 3.1. MSC Amplified in 2D Cultures Express *α*SMA and Inhibit T Cell Proliferation

**Figure 1:**
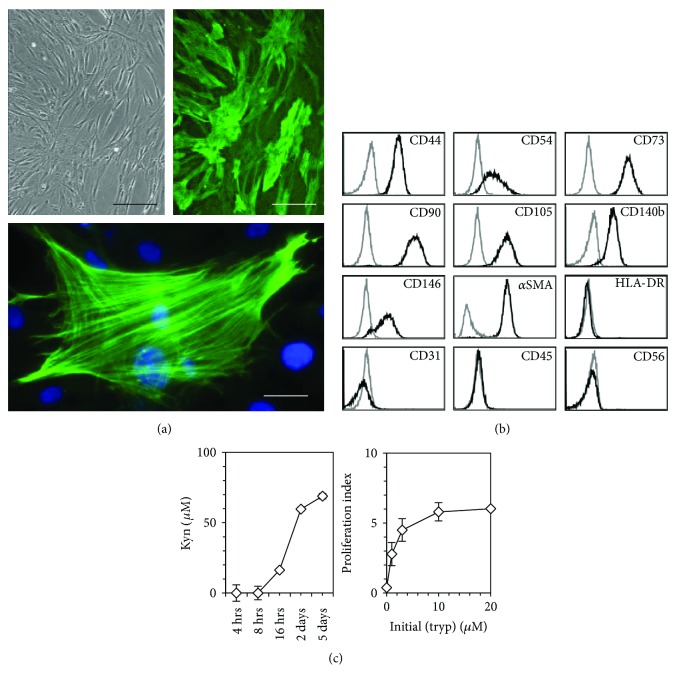
Biological characteristics of MSC cultured in 2D. (a) Amplified cells were fixed in the culture wells with 1% PFA in PBS, permeabilized with ice-cold absolute methanol, and labeled with anti-*α*SMA. *Left panel*: polarized light (scale bar is 150 *μ*m), *right and lower panel*: fluorescence (scale bar is 100 and 20 *μ*m, resp.). Original magnification 10x and 40x for top and lower panels, respectively. (b) Cultures were trypsinated before staining with various antibodies and flow cytometry analysis. Black profile: specific staining, gray profile: isotype control. Data for (a) and (b) are representative of 11 experiments. (c) *Left panel*: kinetics of kyn production by *γ*-IFN-stimulated MSC in IMDM medium (initial (tryp) 80 *μ*M). One single add-on of 500 U/ml of *γ*-IFN was done on day 0. Supernatants were collected at the indicated time points and kyn titers were measured. Data of one experiment, representative of 3. *Right panel*: effect of tryp concentration upon T cell proliferation. Freshly purified, CFSE-labeled T lymphocytes were incubated with a3-28 for 5 days in tryp-free RPMI medium complemented with 0 to 20 *μ*M of tryp, and proliferation was assessed by CFSE dilution. T lymphocyte proliferation is expressed as the inverse of the median CFSE fluorescence observed at the end of the cultures. Data of one experiment, representative of 2. Error bars are SD.

After 3–5 days of culture, BM cell suspensions formed discrete colonies of adherent cells. These colonies kept proliferating and formed a confluent cell layer, which for its vast majority stained positive for *α*SMA (Figure 1(a), top panel). High magnification micrographs showed that *α*SMA was organized in well-defined stress fibers throughout the cytoplasm (Figure 1(a), lower panel). Flow cytometry analysis undertaken on trypsinated cells showed that they expressed CD44, CD54, CD73, CD90, CD105, CD140b (also known as platelet-derived growth factor (PDGF) receptor-*β*), CD146, and *α*SMA, and were negative for HLA-DR, CD31, CD45, CD56 (Figure 1(b)), and CD34 (not shown). Flow cytometry analyses of MSC derived from 11 different donors allowed establishing that *α*SMA-positive cells represented on the mean 94 ± 7.7% of the cells recovered after trypsination of the stroma.

BM-derived MSC were exposed to 500 U/ml *γ*-interferon (*γ*-IFN). After 16 hours, kyn, which was undetectable in fresh medium was identified in MSC supernatants and its concentration increased up to day 5 (Figure 1(c), left panel).

The dependence of T cell proliferation upon the presence of tryp was assessed by stimulating lymphocytes with anti-CD3 and anti-CD28 antibodies immobilized on microbeads (a3-28) in media containing titrated concentrations of tryp. T lymphocytes exhibited a suboptimal proliferation when tryp concentration was <10 *μ*M and could not proliferate in the absence of the amino acid (Figure 1(c), right panel).

The ability of MSC to degrade tryp in the presence of activated T lymphocytes and to inhibit their proliferation was assessed by coculturing lymphocytes stimulated with a3-28 over a MSC stroma. By the end of the 5-day experiment, the titer of tryp averaged 2.5 ± 1.5 *μ*M (*n* = 4) in cocultures containing activated T lymphocytes and MSC, whereas it was 21 ± 2.8 *μ*M in cultures containing activated T lymphocytes exclusively, which is a titer similar to that of RPMI medium incubated for 5 days at 37°C without cells (21 *μ*M of tryp). Kyn, which was undetectable in control medium and in cultures containing activated T lymphocytes exclusively averaged 10 ± 2.9 *μ*M in the cocultures of activated T lymphocytes and MSC. Consistent with the above observation (see Figure 1(c), right panel) and our previous data [18] showing that T lymphocyte proliferation is inhibited when tryp concentration is low, lymphocyte proliferation was efficiently inhibited in the presence of MSC (1.5 ± 1.9 versus 9.4 ± 3.7 CFSE arbitrary proliferation units in the presence and absence of MSC, resp.). Thus the early *γ*-IFN release by activated T lymphocytes was sufficient to activate IDO in MSC to such an extent that the depletion of tryp in the cultures ensuing IDO activation generated an environment that was inhibitory toward T lymphocyte proliferation.

### 3.2. MSC Colonize Collagen Membranes and Express Contractile Proteins

**Figure 2:**
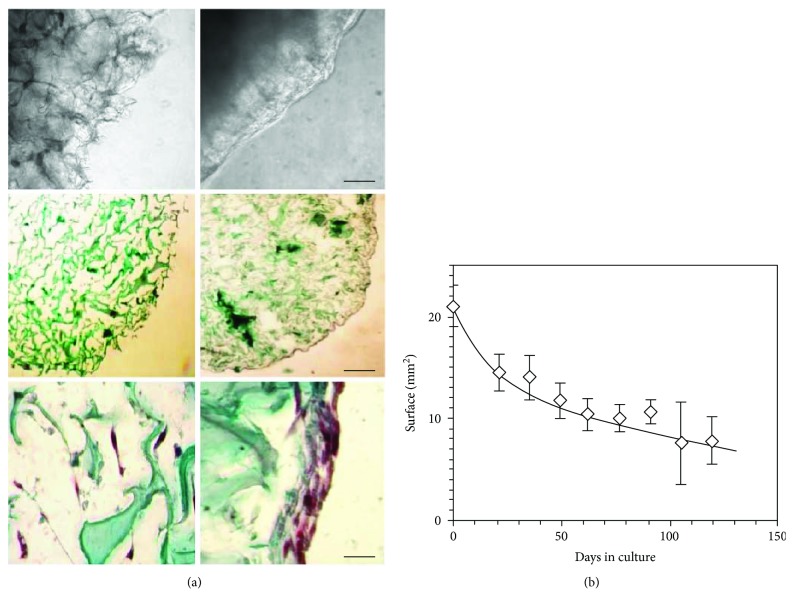
Colonization of collagen membranes by MSC. Collagen membranes were seeded with MSC from passage 1 and maintained in RPMI medium supplemented with HPL for up to 119 days, with the medium changed every 2-3 days. (a) Membranes were observed by light microscopy in the culture vessels (upper panels, scale bar is 500 *μ*m), or after fixation and staining with Goldner's stain (middle and lower panels, scale bars are 500 and 100 *μ*m, resp.). *Upper left panel*: empty membrane; *upper right panel*: MSC-loaded membrane after 9 days of culture. *Middle left panel*: empty membrane section; *middle right panel*: MSC-laden membrane section cultured for 19 days. *Lower panels*: high magnification of a MSC-laden membrane section either in its center (left), or in the periphery (right). (b) Approximate membrane surface expressed in mm^2^ in function of culture time. Data of (a) are of one experiment representative of 5 and data of (b) are the mean ± SD of 19 experiments until day 21 of culture, and of at least 3 experiments thereafter. Original magnifications of (a): higher panels 10x, middle panels 5x, and lower panels 20x.

Four days after 3D culture initiation, the appearance of membranes seeded with MSC was modified: the extended ramifications of the collagen fibers visible by light microscopy prior to cell seeding were no longer detectable. A translucent coating filled the gaps between the fibers and smoothed the surface of the membranes (Figure 2(a), left and right upper panels). After 19 days of culture, membranes were fixed, sliced, and stained with Goldner's stain. This showed that the collagen scaffold of MSC-laden membranes was compressed compared to that of empty membranes (Figure 2(a), middle panels). Moreover, MSC had colonized the inner volume of the supports and had established a multicellular layer on their surface (Figure 2(a), lower panel). The membrane surface, which averaged 21 ± 2 mm^2^ before seeding, decreased to 15 ± 2 mm^2^ after 21 days of culture (*n* = 19 for both time points). Membranes kept decreasing in size thereafter until the end of the experiments (>100 days), albeit at lower pace (Figure 2(b)).

**Figure 3:**
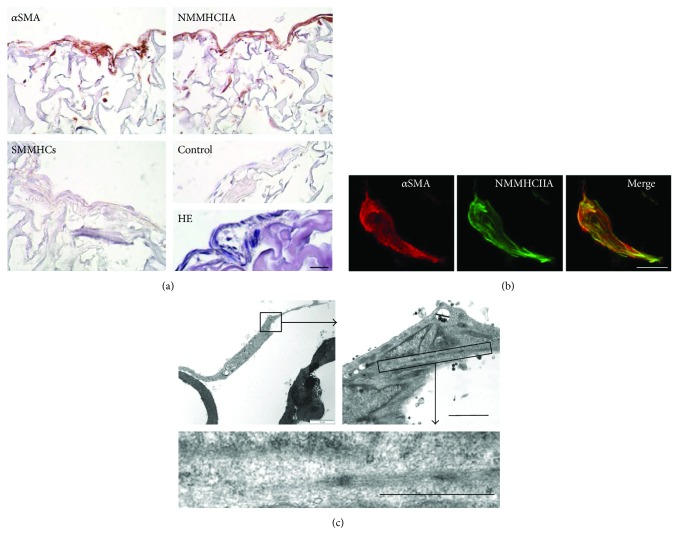
Expression of contractile proteins in MSC seeded in membranes. (a) Membrane sections stained either with anti-*α*SMA, anti-NMMHCIIA, anti-SMMHCs, and control antibody, or HE. (b) MSC-laden membrane stained with anti-NMMHCIIA (left panel), anti-*α*SMA (central panel), and merge picture (right panel) obtained after confocal laser scan microscopy of the paraffin section. Stress fibers positive for both NMMHCIIA and *α*SMA appear yellow on the merge picture. (c) Electronic micrographs with increasing magnification of one MSC located inside a collagen membrane, and cytoplasmic details. Scale bars are 100 *μ*m (a), 10 *μ*m (b), 2 *μ*m, and 1 *μ*m in the right and lower panels of (c), respectively. These data are from cultures harvested 19–21 days post membrane seeding, representative of 3 experiments for (a), 2 for (b), and 2 for (c).

Staining membrane cross sections with anti-*α*SMA, anti-SMMHCs, and anti-NMMHCIIA antibodies showed that cells constituting the surface layer and those residing in the inner space of the membrane expressed *α*SMA and NMMHCIIA (Figure 3(a), upper left and right panels, resp.), but not SMMHCs (Figure 3(a) lower left panel). Confocal microscopy analysis demonstrated that *α*SMA and NMMHCIIA colocalized within the same stress fibers in the MSC grown in collagen membranes (Figure 3(b)). Electronic microscopy identified stress fibers within the cells (Figure 3(c)).

### 3.3. Biological Properties of MSC Cultured in 3-D Collagen Membranes

**Figure 4:**
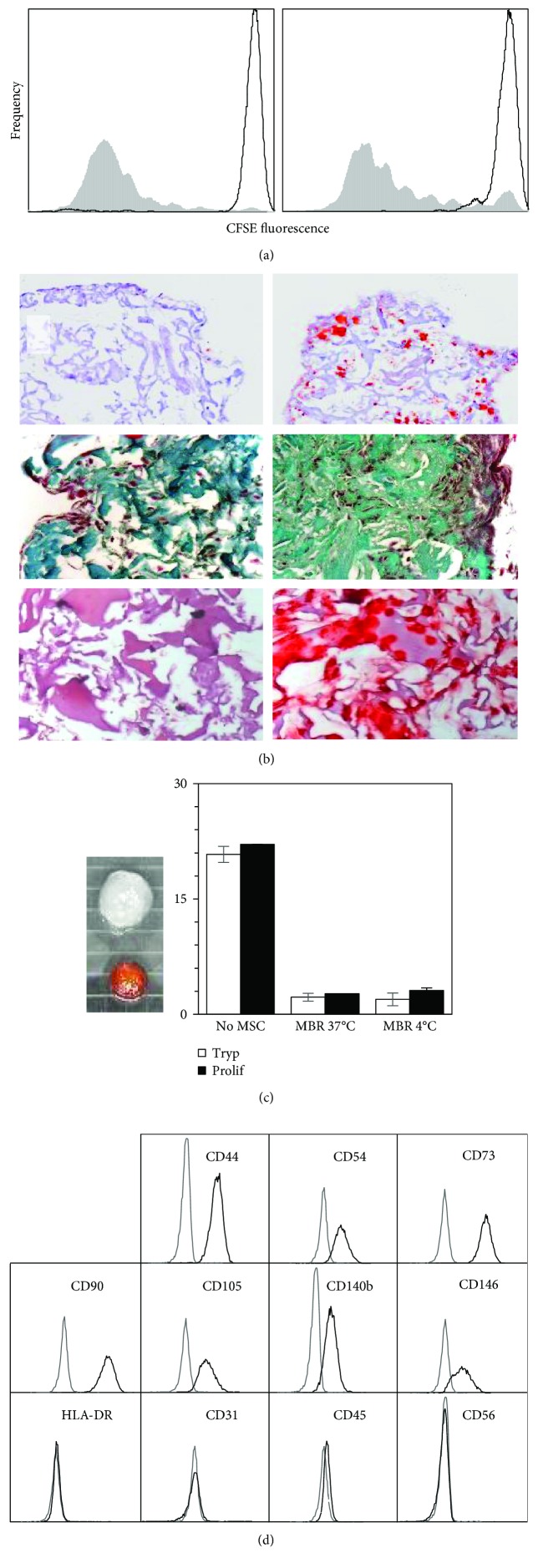
Biological properties of MSC cultured in 3D collagen membranes. (a) Regulation of T lymphocyte proliferation by MSC-laden membranes. T lymphocytes were labeled with CFSE prior to culture, and proliferation was assessed as CSFE fluorescence decrease. *Left panel*: T lymphocyte proliferation in the absence of a membrane and MSC: white profile: quiescent T lymphocytes; gray profile: T cells stimulated with a3-28. *Right panel*: proliferation of T cells stimulated with a3-28 in the presence of membranes: gray profile: empty membrane; white profile: membrane containing MSC. These data are representative of 5 experiments. (b) In vitro differentiation in collagen membranes: *left panels*: control cultures in the presence of HPL only; *upper right*: adipocytic (oil Red O stain); *median right*: chondrocytic (Goldner's stain); *lower right*: osteoblastic (Alizarin R stain) differentiation. Original magnifications are 10x, 20x, and 20x for adipocytic, chondrocytic, and osteoblastic differentiation, respectively. These data are representative of 3 experiments. (c) Biological activities of MSC-laden membranes after storage at 4°C for 0–5 days. *Left*: XTT reduction: an empty membrane (top) and a MSC-laden membrane (bottom) stored for 5 days at 4°C and subsequently exposed to XTT for 2 hours at 37°C are shown. The petri grid consists in 2 × 2 mm squares. *Right*: effect of MSC cocultured with activated T lymphocytes upon tryp degradation and T lymphocyte proliferation: “Tryp”: residual tryp concentration in various supernatants, “Prolif”: T lymphocyte proliferation. “No MSC”: cultures of T cells stimulated with a3-28 in the absence of MSC; “MBR 37°C” and “MBR 4°C”: cocultures of T lymphocytes and membranes seeded with MSC, without or with a previous storage of the latter at 4°C prior to the assay, respectively. The vertical axis refers either to the tryp concentration expressed in micromolar, or to the T cell proliferation expressed as the inverse of the median CFSE fluorescence. These data are representative of 2 experiments. (d) Flow cytometry analyses of surface molecules on MSC after membrane dissolution with collagenase II. The analyses are gated on 7AAD-negative live cells. Flow cytometry data are from one experiment, representative of 6.

MSC-laden membranes prevented T lymphocyte proliferation whereas empty membranes did not (Figure 4(a)). Moreover, MSC seeded in scaffolds and exposed for 3 weeks to various media forcing differentiation toward either adipocytes, chondrocytes, or osteoblasts differentiated along these 3 pathways (Figure 4(b)).

MSC laden in membranes were stored for up to 5 days at 4°C. Their ability to reduce tetrazolium salts (XTT assay), to degrade tryp in the presence of activated T cells, and to prevent T lymphocyte proliferation were examined and compared with membranes that remained in regular cultures at 37°C. MSC-laden membranes stored at 4°C became orange when exposed to XTT, indicating that they were metabolically active, while empty membranes remained white (Figure 4(c), left). The formazan titers recovered in the culture supernatants of MSC-laden membranes that had been stored at 4°C prior to the XTT assay averaged 0.963 ± 0.064 (OD_450_) (mean of day 1–5 of storage at 4°C, *n* = 5), which was similar to the value observed in supernatants of control cultures maintained at 37°C (0.972 (OD_450_)). Moreover, storage at 4°C did not impair the ability of MSC-laden membranes to metabolize tryp in the presence of activated T lymphocytes, and to decrease T cell proliferation (Figure 4(c), right).

Live MSC were released from the membranes by exposure to collagenase II. The number of cells recovered averaged 9.6 ± 2.1 × 10^4^ on day 7 (*n* = 5) and did not vary significantly with culture time (not shown). These cells expressed CD44, CD54, CD73, CD90, CD105, CD140b, and CD146, and were negative for HLA-DR, CD31, CD45, CD56 (Figure 4(d)), and CD34 (not shown). This phenotype was similar to that of MSC grown for the same duration in 2D cultures (see Figure 1(b)). Membrane-derived MSC could be seeded back in 2D cultures where they proliferated and expressed surface markers similar to cells continuously grown on plastic dishes (data not shown).

### 3.4. Mechanical Properties of Collagen Membranes

**Figure 5:**
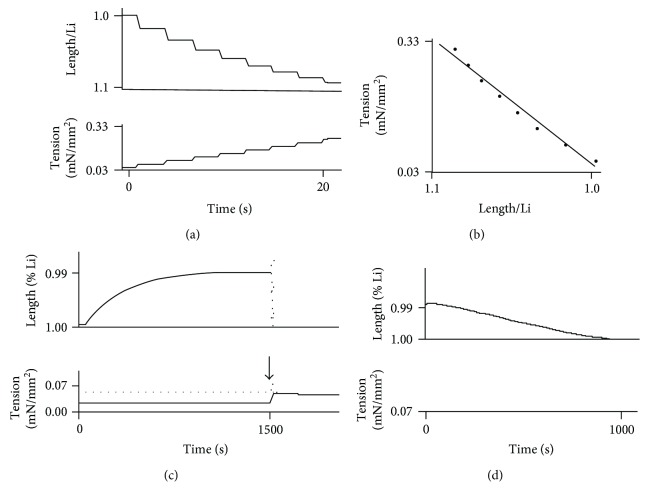
Row data of membrane contractility. Membrane tension is expressed in mN/mm^2^; Li is the membrane length at resting basal tone; time is expressed in seconds (s). (a) Membrane length and tension variations are registered after successive increments by 0.1 mN loading steps, and are shown as a function of time. *Upper panel*: length increase (note that the scale is inverted in the vertical axis). *Lower panel*: tension increase. (b) Computation of the Young modulus using the data generated in (a). The membrane in this example is an empty membrane and its Young modulus is 450 Pa. (c) Effect of 50 mM KCl on a MSC-laden membrane. *Upper panel*: membrane shortening (note that the scale is inverted in the vertical axis on this plot: if membrane shortening increases, membrane length decreases). *Lower panel*: tension generation. The vertical arrow pointing downwards identifies the time point where tension was suddenly increased, shifting the system from isotonic mode to isometric mode. The active tension amplitude equals the total tension represented by the gray dotted line minus the basal tone, which is the lower solid black horizontal profile generated during the isotonic mode. (d) Relaxation of the membrane previously stimulated by KCl by ISDN (NO donor). *Upper panel*: membrane lengthening; *lower panel*: tension observed after the exposure to lSDN. Similar contractile parameters were observed with MSC-laden membranes stimulated by an electric tetanus. These data are representative of 23 experiments for empty membranes and 25 experiments for MSC-laden membranes for the Young modulus determination, 9 and 17 for KCl and tetanus stimulations, respectively, and 10 for ISDN relaxation and 9 for BDM relaxation (see Table 2).

The basic physical properties of membranes, that is, the properties observed without the application of electric or ionic stimulus are presented in Table 1. No significant differences were identified between the various groups in terms of membrane diameter, cross-sectional area (CSA), weight, and basal resting tone (BT). By contrast the mean Young modulus of the empty membranes (569 ± 270 Pascal (Pa)) was found to be significantly smaller to that of MSC-laden membranes (1508 ± 1409 Pa, mean ± SD of 23 and 25 measurements, respectively, *p* = 2 × 10^−4^) (Figures 5(a) and 5(b)).

Contraction and relaxation parameters are presented in Table 2. Membranes without MSC were exposed to electric tetanus because collagen holds converse piezoelectric properties. Piezoelectric effects can be either direct or converse. The direct effect consists in the internal generation of an electric charge resulting from the application of mechanical stress. The converse piezoelectric effect induces the generation of a mechanical strain once the material is exposed to an electric field. Both are observed in solid materials such as crystals, ceramics, collagen, bone, DNA, and various proteins [37, 38]. Applying an electric field to a membrane devoid of MSC induced both a weak shortening and a weak tension of the membrane, which characterizes the converse piezoelectric effect (Table 2(a)). Empty membranes were exposed to KCl because the solvation process could also induce a mechanical tension in collagen molecules. Solvation is a process of attraction and association of molecules of a solvent (here KCl) with molecules or ions of a solute (collagen). Solvation involves hydrogen bonding, ion-dipole, and dipole-dipole attractions or van der Waals forces and is induced when the ionic composition of the solvent is modified [39]. KCl, like tetanus, induced a weak shortening and a weak tension of the empty membranes (Table 2(a)). Once MSC-laden membranes were exposed to tetanus or KCl they shortened and developed an active tension after stimulation that were significantly higher compared with empty membranes (Table 2(a)) and which displayed a very slow kinetics **(**Figure 5(c)). Moreover, MSC-laden membranes stimulated with KCl relaxed after the addition of either isosorbide dinitrate (ISDN) or 2,3-butanedione monoxime (BDM) (Figure 5(d)). Maximum isotonic lengthening (maxVr), negative peak isometric force derivative (−maxdF), and total duration of relaxation induced by both ISDN and BDM were of the same magnitude (Table 2(b)). MSC-laden membranes stimulated by an electric current spontaneously relaxed once the stimulus had stopped and were responsive again after a 10-minute rest. Consequently, the electric stimulation allowed the occurrence of several cycles of contraction-relaxation (data not shown).

## 4. Discussion

In this study we assessed whether the phenotype of MSC derived from human bone marrow was compatible with a pericyte origin, and because several studies reported that myofibroblasts may also originate from pericytes [11, 12], if MSC could differentiate into contractile myofibroblast in vitro. Our investigations comprised phenotypical flow cytometry analyses and various biological assays including cell differentiation assays, MSC-induced T lymphocyte inhibition, and the monitoring of MSC contractility. First, we showed that BM-derived MSC amplified in 2D cultures expressed CD44, CD76, CD90, CD105, CD140b/PDGF receptor-*β*, CD146, and *α*SMA, and were negative for CD45 and CD56 while exhibiting the ability to prevent T lymphocyte proliferation. Such a phenotype, as well as the potent inhibition of T lymphocyte proliferation, has been observed after in vitro amplification of pericytes derived from skeletal muscle [7, 8], thus strongly suggesting that our cells were derived from pericytes as well. Moreover, cell grown in 2D cultures expressed myofibroblast-associated markers such as NMMHCIIA and *α*SMA [40, 41]. One would expect that any cell simultaneously expressing NMMHCIIA and *α*SMA would be able to contract, but cell contractility could not be tested in 2D cultures due to the stiffness of the plastic supporting the stroma. Second, we showed that MSC survived for extended periods in collagen membranes and maintained a potent metabolic activity as documented by the XTT assay. However, the direct comparison of the metabolic activity developed by cells grown in 2D and 3D cultures could not be established using the XTT assay because the formazan salt released by the metabolizing cells was partially trapped in the membranes (coloring them in orange), and could not be quantitatively recovered for the densitometric analyses thus precluding an unbiased comparison with 2D cultures. Additional bioassays (see below) confirmed that MSC were fully functional while residing in the membranes, even after extended storage at 4°C. The array of biological markers expressed by the cells in 3D cultures, including NMMHCIIA and *α*SMA, was identical to that of the 2D cultures. Quite remarkably these contractile molecules were functional as evidenced by the fact that MSC-laden membranes exposed to the classical modes of sarcomeric and nonsarcomeric muscle stimulation readily contracted. Third, while the majority if not the totality of cells cultured with HPL expressed *α*SMA (see Figure 1(b)), our investigations showed that MSC laden in the membranes were simultaneously endowed with the ability to differentiate into chondrocytes, osteoblasts, or adipocytes, and prevented allogenic T lymphocyte proliferation. Thus, globally our data indicate that BM-derived MSC amplified with HPL most probably descended from pericytes, and spontaneously differentiate, when seeded in collagen membranes, into functional myofibroblasts while conserving phenotypic and functional features traditionally attributed to MSC.

If, in vivo, myofibroblasts exhibit the integrality of these features as well, not only would they help injury resolution via matrix synthesis and cellular contraction [22] but also by contributing directly to tissue reconstruction, and by preventing chronic inflammation via the silencing of delayed T cell activation. This hypothesis is consistent with the work of Pinchuk et al. [23], which showed that colonic myofibroblasts of human guts efficiently inhibit T cell proliferation and may play a prominent role in the establishment and sustenance of mucosal intestinal tolerance. Thus it may be interesting to reassess the overall contribution of myofibroblasts upon systemic immune tolerance.

Some studies have indirectly assessed the retractile properties of MSC differentiated into myofibroblasts (for review see [22]). BM-derived MSC expressing *α*SMA have been reported to retract in collagen gels as determined by comparing gel size before and after incubation with TGF-*β* or PDGF using photographs [42] or a microscope micrometer [43]. However in order to claim that a tissue is contractile one has to show that it can contract under the action of a stimulus that is generally electric or (bio)chemical, and that it can relax when exposed to appropriate drugs, such as BDM that targets actin-myosin bridges or ISDN that decreases intracellular calcium. This dual process of contraction-relaxation has to be reversible, that is, it can be applied several times to the same preparation. Moreover, one has to provide data concerning the strength/tension developed by the preparation during the exposition to the stimulus using transducers monitoring the entire process in real time. Although the studies quoted above [42, 43] demonstrated that cells retracted, they did not quantify the cell mechanical power developed during the process of contraction, as measured in the present work.

Other studies quantified the force developed by myofibroblasts (but not MSC) trapped in gels, or used substrates of defined compliance to establish the correlation between support stiffness and myofibroblast maturation [44–47], but none measured the Young modulus of matrices before and after cell colonization, nor assessed the impact of classic modes of sarcomeric and nonsarcomeric muscle stimulation such as electric tetanus and KCl upon MSC/myofibroblast contraction.

In this study, we showed that the Young modulus of collagen membranes significantly increased when the structures were infiltrated with MSC. This indicates that cells firmly bound to the collagen membranes, as confirmed by the necessity to use collagenase to release them from the scaffold. TGF-*β*, present in biologically active concentrations in HPL [3] and known to enhance cell attachment to collagen [28, 48, 49], is certainly involved in this process. Moreover, the significant decrease of membrane size observed during culture (see Figure 2(b)) may be provoked together by TGF-*β* and the increase of membrane stiffness associated with membrane colonization. Both are known to favor myofibroblast contraction [44–46].

To validate our study, we had to identify the active mechanical properties of the membranes devoid of MSC. These are the converse piezoelectricity and solvation, which can occur when empty membranes are submitted to an electric field, or are soaked in high salt concentration, respectively [37, 38]. Hopefully enough, the mechanical tension induced on empty membranes by an electric field or a solvation process were significantly lower than those observed when MSC-loaded membranes were exposed to the stimuli. These findings, plus the observation that BDM and ISDN induced membrane relaxation, further confirmed that MSC-laden membrane contractions were produced by cells that were metabolically active and did not result from chemical or physical artifacts.

The fine mechanics of nonmuscular contractile biological systems has been investigated in the human placenta [36, 50, 51]. Placental myofibroblasts are located in the stem villi [52], exhibit a low isometric tension [49–51], and have a slow shortening velocity. They also exhibit a low myosin ATPase activity [53–55]. This is coherent with the observation that the dominant molecular motor in placental myofibroblasts is the nonmuscle myosin IIA [40, 41] whose molecular kinetics are dramatically slow [36, 55]. MSC-laden membrane stimulation lead to a similarly slow shortening velocity, which is consistent with the observation that nonmuscle myosin IIA, but not smooth muscle myosin, was detectable in the cells laden in membranes.

## 5. Conclusion

This work showed that MSC derived from human BM and cultured in collagen membranes supplemented with HPL expressed a phenotype that is compatible with a pericyte origin. Moreover, MSC spontaneously differentiated in the collagen scaffold into contractile myofibroblasts, which were simultaneously able to control T lymphocyte proliferation and to differentiate further into various mesenchymatous lineages. Our data suggest that myofibroblasts are not exclusively involved in tissue healing as initially sought, but also participates to inflammation shutdown and perhaps, due to their broad distribution within the body, to systemic self-tolerance as well. In addition MSC-laden collagen membranes, due to the extended survival of MSC in these structures, can be shipped to distant destinations without altering the biological potency of the cells, therefore representing a versatile and promising therapeutic tool to control local inflammation or favor the reconstruction of various tissues after injury.

## Figures and Tables

**Table 1:** Basic physical properties of the membranes.

	Tetanus		KCl		*p*
	No MSC (*n* = 10)	MSC (*n* = 17)	No MSC (*n* = 10)	MSC (*n* = 9)	
Diameter (mm)	6.1 ± 0.7	5.4 ± 1.1	6.1 ± 0.2	5.8 ± 0.9	0.09
CSA (mm^2^)	2.2 ± 0.6	2.4 ± 0.8	2.3 ± 1.1	2.3 ± 0.2	0.96
Weight (mg)	27.7 ± 10.1	22.5 ± 6.9	29.2 ± 14.9	25.3 ± 5.5	0.33
Basal tone (mN/mm^2^)	0.037 ± 0.013	0.028 ± 0.013	0.033 ± 0.017	0.032 ± 0.005	0.51

Mean values ± SD of diameter, cross-sectional area of membranes (CSA), weight, and basal tone are shown. No MSC: empty collagen membrane; MSC: MSC-laden membrane; *p*: *p* value obtained from variance analysis performed on the 4 types of membranes. No significant difference is observed for the 4 parameters analyzed.

**Table:** (a) Contraction

	Tetanus		*p*	KCL		*p*
	No MSC (*n* = 12)	MSC (*n* = 17)		No MSC (*n* = 6)	MSC (*n* = 9)	
ΔLmax (%)	0.003 ± 0.003	0.013 ± 0.009	<0.001	0.003 ± 0.003	0.010 ± 0.003	<0.05
Active tension (mN/mm^2^)	0.009 ± 0.009	0.045 ± 0.035	<0.001	0.007 ± 0.008	0.034 ± 0.014	<0.05

**Table:** (b) Relaxation

	ISDN (*n* = 10)	BDM (*n* = 9)	*p*
Max Vr (mm/s)	0.048 ± 0.023	0.037 ± 0.023	0.35
Isot. relax. time (s)	980 ± 774	972 ± 433	0.98
−Max dF (mN/s)	0.48 ± 0.05	0.41 ± 0.30	0.64
Isom. relax. time (s)	1330 ± 877	1750 ± 901	0.45

(a) Maximum amplitude of isotonic shortening (ΔLmax) and *p* values comparing membranes with and without MSC exposed to either tetanus or KCl are shown. (b) Maximum relaxation velocity (Max Vr), time to isotonic relaxation (Isot. relax. time), negative peak of isometric relaxation (−max dF), and time to isometric relaxation (Isom. relax. time) are shown. No significant statistical differences were observed between ISDN and BDM for the 4 parameters of relaxation. (*n*) refers to the number of duplicates undertaken.
